# Integrative Serum Metabolic Fingerprints Based Multi‐Modal Platforms for Lung Adenocarcinoma Early Detection and Pulmonary Nodule Classification

**DOI:** 10.1002/advs.202203786

**Published:** 2022-10-18

**Authors:** Lin Wang, Mengji Zhang, Xufeng Pan, Mingna Zhao, Lin Huang, Xiaomeng Hu, Xueqing Wang, Lihua Qiao, Qiaomei Guo, Wanxing Xu, Wenli Qian, Tingjia Xue, Xiaodan Ye, Ming Li, Haixiang Su, Yinglan Kuang, Xing Lu, Xin Ye, Kun Qian, Jiatao Lou

**Affiliations:** ^1^ Department of Laboratory Medicine Shanghai General Hospital Shanghai Jiao Tong University School of Medicine Shanghai 200080 P. R. China; ^2^ Department of Laboratory Medicine Shanghai Chest Hospital Shanghai Jiao Tong University School of Medicine Shanghai 200030 P. R. China; ^3^ State Key Laboratory for Oncogenes and Related Genes School of Biomedical Engineering Institute of Medical Robotics and Med‐X Research Institute Shanghai Jiao Tong University Shanghai 200030 P. R. China; ^4^ State Key Laboratory for Oncogenes and Related Genes Division of Cardiology Renji Hospital Shanghai Jiao Tong University School of Medicine Shanghai 200127 P. R. China; ^5^ Department of Thoracic Surgery Shanghai Chest Hospital Shanghai Jiao Tong University School of Medicine Shanghai 200030 P. R. China; ^6^ Department of Laboratory Medicine The Third Hospital of Hebei Medical University Shijiazhuang 050051 P. R. China; ^7^ School of Medicine Jiangsu University Zhenjiang 212013 P. R. China; ^8^ Department of Radiology Shanghai Chest Hospital Shanghai Jiao Tong University School of Medicine Shanghai 200030 P. R. China; ^9^ Department of Radiology Shanghai Institute of Medical Imaging Zhongshan Hospital Fudan University Shanghai 200032 P. R. China; ^10^ Department of Laboratory Diagnostics The First Affiliated Hospital of USTC Division of Life Sciences and Medicine University of Science and Technology of China Hefei Anhui 230001 P. R. China; ^11^ Gansu Academic Institute for Medical Research Gansu Cancer Hospital Lanzhou Gansu 730050 P. R. China; ^12^ Department of A. I. Research Joint Research Center of Liquid Biopsy in Guangdong, Hong Kong, and Macao Zhuhai Guangdong 519000 P. R. China; ^13^ Department of Product Development Joint Research Center of Liquid Biopsy in Guangdong, Hong Kong, and Macao Zhuhai Guangdong 519000 P. R. China

**Keywords:** deep learning, lung adenocarcinoma, metabolomics, multi‐modal, pulmonary nodule

## Abstract

Identification of novel non‐invasive biomarkers is critical for the early diagnosis of lung adenocarcinoma (LUAD), especially for the accurate classification of pulmonary nodule. Here, a multiplexed assay is developed on an optimized nanoparticle‐based laser desorption/ionization mass spectrometry platform for the sensitive and selective detection of serum metabolic fingerprints (SMFs). Integrative SMFs based multi‐modal platforms are constructed for the early detection of LUAD and the classification of pulmonary nodule. The dual modal model, metabolic fingerprints with protein tumor marker neural network (MP‐NN), integrating SMFs with protein tumor marker carcinoembryonic antigen (CEA) via deep learning, shows superior performance compared with the single modal model Met‐NN (*p* < 0.001). Based on MP‐NN, the tri modal model MPI‐RF integrating SMFs, tumor marker CEA, and image features via random forest demonstrates significantly higher performance than the clinical models (Mayo Clinic and Veterans Affairs) and the image artificial intelligence in pulmonary nodule classification (*p* < 0.001). The developed platforms would be promising tools for LUAD screening and pulmonary nodule management, paving the conceptual and practical foundation for the clinical application of omics tools.

## Graphical abstract

A multiplexed assay is developed on an optimized nanoparticle‐based laser desorption/ionization mass spectrometry platform for the sensitive detection of serum metabolic fingerprints (SMFs). Integrative SMFs based multi‐modal platforms are constructed for lung adenocarcinoma early detection by deep learning. The strategy is demonstrated to be practically feasible to aid the existing diagnostic approaches of lung cancer.

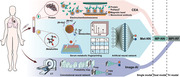

## 1 Introduction

Lung cancer, with 2.22 million new cases and 1.79 million deaths globally in 2020,^[^ ^1^ ^]^ is a complex heterogeneous malignant tumor.^[^ ^2^ ^]^ Lung adenocarcinoma (LUAD) is the most popular histological subtype of non‐small cell lung cancer, which constitutes about 50% of lung malignancies.^[^ ^3^ ^]^ Although annual radiologic screening of LUAD by low‐dose computed tomography (LDCT) is suggested for populations of high risk, the management of LDCT‐detected pulmonary nodule, the main manifestation of early stage LUAD, is challenging with a false positive rate of 96%.^[^ ^4^ ^]^ Clinical nodule assessment tools (e.g., Mayo Clinic and Veterans Affairs (VA) models^[^ ^5^ ^]^) as well as computer‐aided diagnosis methods^[^ ^5b^ ^]^ have been widely used, whereas the performance is limited due to lack of tumor‐specific biological information. Therefore, there is an unmet need for complementary biomarkers for early diagnosis of LUAD and accurate classification of pulmonary nodule.

Emerging biomarkers for cancer early diagnosis vary from cellular to molecular level. At the cellular level, circulating tumor cells (CTCs), as a metastatic relapse and prognostic marker, may help monitor the progression of lung cancer. Still, CTCs show limited sensitivity of 70% and require hours for antibody recognition and enrichment, making it infeasible for large‐scale cancer screening.^[^ ^6^ ^]^ At the molecular level, omics tools, including genomics,^[^ ^7^ ^]^ proteomics,^[^ ^8^ ^]^ and metabolomics,^[^ ^9^ ^]^ inform clinical decision‐making for cancer diagnosis and guide precision medicine. Notably, metabolic analysis, which is closer to disease phenotypes, measures the end products of biological activities and represents a promising tool for the detection of early‐stage cancer.

As the state‐of‐art analytical tools for metabolomics, mass spectrometry (MS) and nuclear magnetic resonance (NMR) dominate metabolic analysis. NMR measures atomic species by the electromagnetic interaction between atom spins and magnetic fields,^[^ ^10^ ^]^ which requires a long detection time owing to the relaxation.^[^ ^10a^ ^]^ In contrast, MS directly records molecular species by fragmentation spectrum and mass‐to‐charge ratio (*m*/*z*), affording advanced molecular identification ability.^[^ ^11^ ^]^ Current MS based metabolic analysis is usually performed in a targeted and expensive manner, which detects a limited amount of metabolites and requires tedious pretreatment (e.g., derivatization and desalination) and long assay time (∼12 h per sample). In contrast, non‐targeted laser desorption/ionization (LDI) MS affords strengths of high‐throughput, no pretreatment, and rapid analysis. Whereas, LDI MS usually demands organic matrices for energy and ion/electron transfer during the desorption/ionization process, decreasing the detection accuracy due to the interference in the low mass range and limited selectivity/sensitivity.^[^ ^11^, ^12^ ^]^ With the development of nanomaterial,^[^ ^11^, ^13^ ^]^ a nanoparticle based laser desorption/ionization mass spectrometry (NPLDI MS) can improve the charge transfer and reduce heat dissipation for photon‐induced desorption/ionization of analytes.^[^ ^11^, ^12^ ^]^ Notably, the energy absorption is performed without forming cluster ions which degenerate the biological signals, making it a potential solution for LDI MS.^[^ ^11a^ ^]^

The development and occurrence of diseases, especially cancer, consist of complicated biological mechanisms, in which single modal data is insufficient for analyzing the pathogenic factors. Multi‐omics analyses will not only be able to reveal the complex mechanisms underlying cancer progression and development,^[^ ^14^ ^]^ but also have been proposed as the key to promoting precision medicine in the clinic, such as diagnostic/therapeutic biomarker discovery,^[^ ^15^ ^]^ treatment response evaluation,^[^ ^16^ ^]^ and survival prediction.^[^ ^17^ ^]^ Particularly, a recent prospective and interventional study demonstrated that blood tests with positron emission tomography‐computed tomography can localize several types of cancers safely and precisely for individuals who were not previously known to have cancer,^[^ ^18^ ^]^ proofing the clinical potential of integrating biological biomarkers with radiological features for early cancer screening.

The success of multi‐modal applications depends on tailored data science models. In our work, the integrative serum metabolic fingerprints (SMFs) are defined as SMFs, known for high‐dimensionality and sparsity,^[^ ^12b^ ^]^ in combination with the protein tumor marker and image features, making it unstructured data instead of structured data. Due to the nonlinear transformation and maximum utilization of unstructured data,^[^ ^19^ ^]^ deep learning methods have been successfully applied in multi‐modal data analysis, such as the infusion of transcriptomics and microscopy/electrophysiology data for neuronal cells.^[^ ^20^ ^]^ Yet, deep learning for modeling requires careful structure design and parameter tuning, making a tailored deep learning model for integrative SMFs in demand.

Herein, we developed a multiplexed assay on an optimized NPLDI MS platform for the direct metabolic analysis of patients' sera. The NPLDI MS platform can directly trap metabolites from complex biosamples, allowing for a high‐throughput and high sensitive/selective collection of metabolic fingerprints. By combining SMFs with clinical indexes (protein tumor marker, carcinoembryonic antigen (CEA) and image features), we achieved both early diagnosis of LUAD and accurate classification of pulmonary nodule with artificial intelligence strategies toward multi‐modal recognition. The integrative SMFs model may represent a revolution in screening of early‐stage cancer and contribute to improving health care, not limited to LUAD.

## 2 Results

### 2.1 Study Design

**Figure 1:**
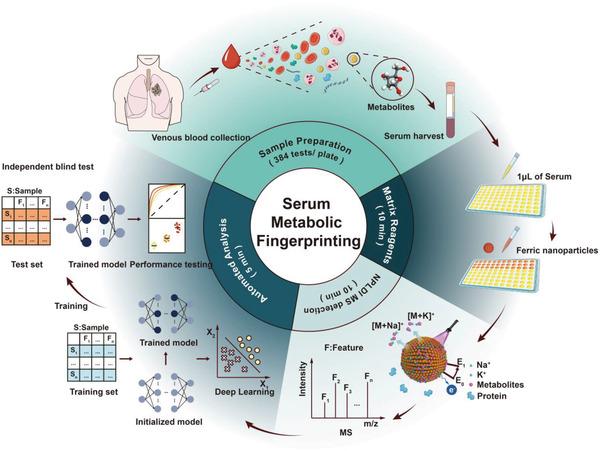
Overall experimental schematic of nanoparticle based laser desorption/ionization mass spectrometry (NPLDI MS). 1 µL of native serum was incubated with ferric nanoparticles without pretreatment and directly analyzed by laser desorption/ionization mass spectrometry (LDI MS) to record Na+ and K+ adducted signals. The deep learning method was used to construct diagnostic models based on the serum metabolic fingerprints. Blind test was conducted with the same protocol to evaluate the performance of the diagnostic models.

The workflow of the study, including sample preparation, matrix reagents loading, NPLDI MS detection, and automated analysis, is presented in **Figure** **1**. To determine LUAD specific SMFs and develop the integrative SMFs based diagnostic model, we employed NPLDI MS to record SMFs of treatment‐naïve LUAD patients (*n* = 958) and clinically relevant controls (*n* = 1318) as shown in Table S1, Supporting Information. Furthermore, from the above participants, a total of 480 samples with pulmonary nodule on LDCT images were selected to construct and evaluate the multi‐modal model for nodule classification (Figure S1, Supporting Information).

### 2.2 Extraction of SMFs

Ferric nanoparticles (Figure S2A, Supporting Information) were chosen as the matrix of NPLDI MS for the rough surface, strong absorption of light, good water dispersity, and the negative‐charged surface. For the rough surface indicated by scanning electron microscopy (SEM, Figure S2B, Supporting Information), it could assist the size‐selective trapping of metabolites.^[^ ^13e^ ^]^ For the strong absorption of light indicated by the ultraviolet‐visible spectrum (Figure S2D, Supporting Information), it could help the transfer of laser energy during desorption/ionization process. In addition, the ferric nanoparticles also display the good water dispersity for evenly mixing the suspension without precipitation (dynamic light scattering analysis, Figure S2E, Supporting Information) and the negative‐charged surface (zeta potential, Figure S2F, Supporting Information) for cation adduction.

We further evaluated the analytical performance of the NPLDI MS from the aspects of sensitivity, protein/salt tolerance, and stability. For sensitivity, NPLDI MS with ferric nanoparticles could detect trace amounts of analytes in biofluids with a detection limit as low as ∼pmol (Table S2, Supporting Information). For protein/salt tolerance, NPLDI MS with ferric nanoparticles successfully identified the molecular peaks from the highly concentrated protein (Figure S3A, Supporting Information) and salt solutions (Figure S3B,C, Supporting Information). For detection stability, we performed two experiments to evaluate the NPLDI MS platform: 1) repeated experiments of the same sample with the same conditions; 2) repeated experiments of the same sample with ferric nanoparticles from different batches. In the first experiment, we performed NPLDI MS detection of glucose, valine, and lysine with the concentration of 1 ng nL^−1^ in three technical replicates, and observed a coefficient of variation (CV) of 2.72%, 10.83%, and 8.06% for glucose, valine, and lysine in sodium adducts, respectively (Figure S4, Supporting Information). In the second experiment, we performed the NPLDI MS detection using the ferric nanoparticles from three batches (average size of 332.5, 313.0, and 332.1 nm, Figure S5A–C, Supporting Information), and observed a CV < 10% for glucose, valine, and lysine in sodium adducts (Figure S5D–F, Supporting Information). Therefore, the property of high sensitivity, protein/salt tolerance, and stability make ferric nanoparticles the suitable matrix for LDI MS detection.

We then built the serum metabolic database using the high‐throughput NPLDI MS analysis. In total, there existed ∼125 000 data points in the raw MS results per sample, and the *m*/*z* signals were attained with total ion counts (referred to the sum of the peak intensity for every sample) of ∼8.29 × 10^6^ at the low mass range of 100∼400 Da (Figure S6A, Supporting Information). Notably, the SMFs, which consisted of 2316 *m*/*z* signals, were extracted from raw MS results after data preprocessing (Figure S6B, Supporting Information), serving as the input data for diagnosis use.

### 2.3 SMFs Based Single and Dual Modal Models for LUAD Diagnosis

**Figure 2:**
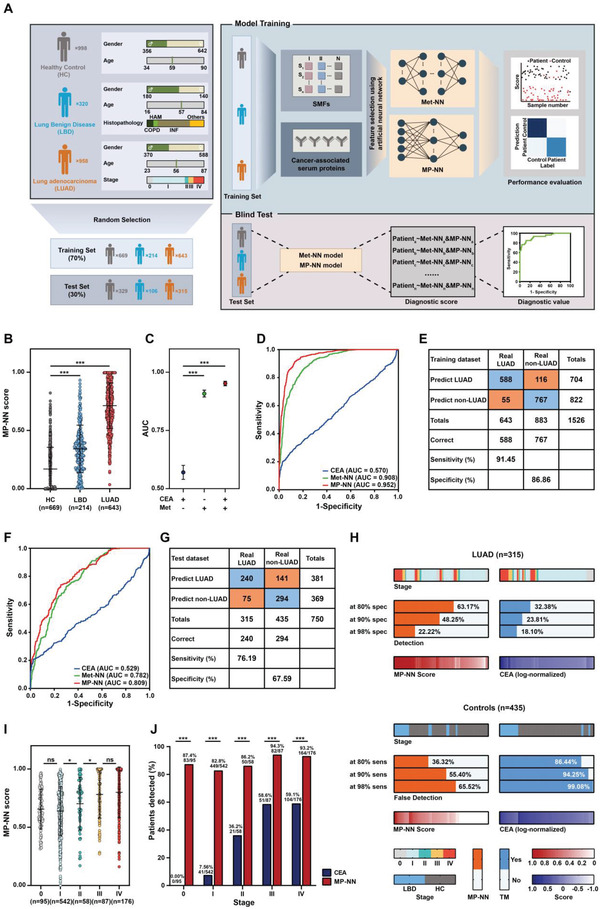
Development and blind test of serum metabolic fingerprints (SMFs) based lung adenocarcinoma (LUAD) diagnostic model. A) Schematic overview of the deep learning approach used to develop and validate the SMFs based integrated LUAD diagnostic model. B) Score of the MP‐NN identified in the training cohort. *p*‐values were calculated using a Wilcoxon test. Error bars refer to interquartile. C) Area‐under‐curve (AUC) for individual parameters in the training cohort. *p*‐values were calculated using a DeLong test. Error bars refer to 95% confidence intervals (CIs). D) Receiver operating characteristic curve (ROC) of the individual parameters in the training cohort. E) Confusion tables of binary results of the MP‐NN model in the training cohort. F) ROC of the individual parameters in the test cohort. G) Confusion tables of binary results of the MP‐NN model in the test cohort. H) Detection rates of MP‐NN and carcinoembryonic antigen (CEA) at different specificity in the test cohort. I) MP‐NN score levels summarized by stage in the whole LUAD cohort. *p*‐values were calculated using a Chi‐square test. Error bars indicate interquartile. J) MP‐NN detection rates summarized by stage in the whole LUAD cohort. *p*‐values were calculated using a Chi‐square test. *p < 0.05, and *** *p* < 0.001.

In order to validate the diagnostic capacity of SMFs for clinical use, we randomly split the enrolled samples into a training cohort of *n* = 1526 (*n* = 669/214/643; HC/LBD/LUAD) and a non‐overlapped test cohort of *n* = 750 (*n* = 329/106/315; HC/LBD/LUAD) with well‐matched age and sex (*p* > 0.05) (**Figure** **2**A). Notably, the samples were mainly from early‐stage LUAD, with stage 0 – II cancers comprising 71.2% (458/643) and 75.2% (237/315) of the total patients in the training and test cohort, respectively (Table S1, Supporting Information).

The modeling of MS big data in serum samples plays a critical role in the clinical application to obtain accurate performances. The results of t‐SNE showed no clustering in the first two principal components, indicating the hardly indistinguishable nature of SMFs by naïve machine learning methods (Figure S7A,B, Supporting Information). We applied conventional machine learning for LUAD diagnosis with SMFs plus protein tumor marker CEA to verify the hardly indistinguishable nature. We performed linear models, which include logistic regression (LR), elastic net (EN), and least absolute shrinkage and selection operator (LASSO), and nonlinear models, which include support vector machine (SVM) and random forest (RF), for the binary classification. For linear models, the area‐uder‐curve (AUC) of blind test achieved 0.659/0.576/0.570 for LASSO/EN/LR, respectively (Figure S7C, Supporting Information). For nonlinear models, we obtained AUCs of 0.746 and 0.657 for RF and SVM, respectively (Figure S7D, Supporting Information).

Then, we turned to deep learning for building the diagnostic models. We named the model based on SMFs as METabolic Neural Network (Met‐NN) and the model based on SMFs plus protein tumor marker as Metabolic fingerprints with Protein tumor marker Neural Network (MP‐NN, Figure 2A, Figure S7E, Supporting Information). The MP‐NN score showed an increasing trend from HC to LUAD, with significantly higher scores in patients with LUAD than those in HC and LBD (*p* < 0.001, Figure 2B). The MP‐NN model showed excellent capacity for distinguishing LUAD patients from HC and LBD patients for the training set (Figure 2C–E). As shown in **Table** **1**, the obtained AUC value for LUAD versus HC/LBD was 0.952 (95% confidence interval (CI) 0.942–0.961), correlating to a sensitivity/specificity combination of 91.45%/86.86%, which was significantly higher than the AUC of CEA (0.570, 95% CI 0.540–0.600; *p* < 0.001) and the single modal model Met‐NN (0.908, 95% CI 0.894–0.923; *p* < 0.001). Importantly, the MP‐NN model demonstrated better performance for the detection of early‐stage LUAD patients (stage 0‐II) from HC/LBD patients than CEA (*p* < 0.001) and the Met‐NN (*p* < 0.001) and even for the detection of patients with adenocarcinoma in situ (stage 0) (Table 1).

**Table 1:** MP‐NN performance for lung adenocarcinoma (LUAD) diagnosis

Characteristics	Threshold	AUC (95% CI)	Sensitivity [%]	Specificity [%]	PPV [%]	NPV [%]	Accuracy [%]	*p*1 value^a)^	*p*2 value^b)^
	**Training set**								
**AD (Stage 0‐IV)**									
CEA	5 ng mL^−1^	0.570 (0.540–0.600)	23.79	92.75	70.51	62.57	63.70	/	/
Met‐NN	0.521	0.908 (0.894–0.923)	86.63	78.94	74.97	89.02	82.18	<0.001	<0.001
MP‐NN	0.433	0.952 (0.942–0.961)	91.45	86.86	83.52	93.31	88.79	<0.001	
**AD (Stage 0‐II)**									
CEA	5 ng mL^−1^	0.527 (0.494–0.561)	9.61	92.75	40.74	66.42	64.35	/	/
Met‐NN	0.521	0.908 (0.892–0.923)	86.68	78.94	68.10	91.95	81.58	<0.001	<0.001
MP‐NN	0.433	0.943 (0.931–0.955)	89.96	86.86	78.03	94.34	87.92	<0.001	
**AD (Stage 0)**									
CEA	5 ng mL^−1^	0.679 (0.613–0.745)	0.00	92.75	0.00	92.54	86.30	/	/
Met‐NN	0.521	0.916 (0.886–0.946)	92.42	78.94	24.70	99.29	79.87	<0.001	<0.001
MP‐NN	0.433	0.945 (0.925–0.965)	89.39	86.86	33.71	99.10	87.04	<0.001	
	**Test set**								
**AD (Stage 0‐IV)**									
CEA	5 ng mL^−1^	0.529 (0.485–0.573)	20.32	97.47	85.33	62.81	65.07	/	/
Met‐NN	0.521	0.782 (0.750–0.814)	76.19	63.45	60.15	78.63	68.80	<0.001	0.004
MP‐NN	0.433	0.809 (0.779–0.839)	76.19	67.59	62.99	79.67	71.20	<0.001	
**AD (Stage 0‐II)**									
CEA	5 ng mL^−1^	0.575 (0.528–0.622)	7.59	97.47	62.07	65.94	65.77	/	/
Met‐NN	0.521	0.775 (0.741–0.810)	75.53	63.45	52.96	82.63	67.71	<0.001	0.463
MP‐NN	0.433	0.782 (0.748–0.817)	71.73	67.59	54.66	81.44	69.05	<0.001	
**AD (Stage 0)**									
CEA	5 ng mL^−1^	0.636 (0.524–0.748)	0.00	97.47	0.00	93.60	91.38	/	/
Met‐NN	0.521	0.794 (0.726–0.862)	75.86	63.45	12.15	97.53	64.22	0.027	0.002
MP‐NN	0.433	0.843 (0.789–0.898)	82.76	67.59	14.55	98.33	68.53	0.003	

AUC = area under curve; NPV = negative predictive value; PPV = positive predictive value; CI = confidence interval.

^a)^ The *p*1 values indicate the statistical significance for the differences of AUC as compared with traditional tumor marker CEA.

^b)^ The *p*2 values indicate the statistical significance for the differences of AUC between Met‐NN and MP‐NN.

We further validated the MP‐NN model in the independent test set. Consistent results were acquired with an increasing MP‐NN score trend from HC to LUAD (*p* < 0.001; Figure S6C, Supporting Information) and comparable diagnostic performance (AUC of 0.809, correlating to a sensitivity of 76.19% and specificity of 67.59%) within the training cohort (Table 1). We attributed the success of MP‐NN to nonlinear transformation and maximum utilization of unstructured data. For nonlinear transformation, LR, LASSO, and EN only achieved AUCs of 0.570, 0.659, and 0.576 in blind test set, respectively (Figure S7C, Supporting Information). In contrast, MP‐NN obtained a significant AUC of 0.809 (*p* < 0.0001 compared with LASSO, EN, and LR), indicating the superiority of nonlinear transformation. For maximum utilization of unstructured data, MP‐NN achieved a significant AUC of 0.809 in blind test set, while RF and SVM only had AUCs of 0.764 and 0.658 (*p* < 0.0001 compared with deep learning method) (Figure S7D, Supporting Information), indicating the superiority of its better utilization of unstructured data (multiple inputs for deep neural network).

The MP‐NN model outperformed the traditional tumor marker CEA (Figure 2F,G; Figure S6D, Supporting Information) in the test cohort. In addition, the detection rate of MP‐NN model in the LUAD group was higher than that of CEA with specificity of 80%, 90%, and 98%, and the false positive rate was lower with sensitivity of 80%, 90%, and 98% (Figure 2H). We also found a correlation between MP‐NN scores and LUAD clinical stage (Figure 2I). Particularly, MP‐NN detected 87.4%, 82.8%, 86.2%, 94.3%, and 93.2% of patients with stage of 0, I, II, III, and IV LUAD, respectively (Figure 2J), demonstrating that MP‐NN score may serve as a direct indicator of tumor burden and may show utility in treatment monitoring and early detection of cancer relapse (Figure S6E,F, Supporting Information). Besides, we further performed a permutation test to evaluate the overfitting effects of MP‐NN (Figure S8, Supporting Information). Specifically, the distribution of the AUC in the blind test set demonstrated no overfitting effect significantly (*p* < 0.05). The AUC was calculated with the uninformative data obtained from random permutation, which has been universally employed to estimate overfitting.^[^ ^21^ ^]^

### 2.4 Performance of MP‐NN for Pulmonary Nodule Classification

**Figure 3:**
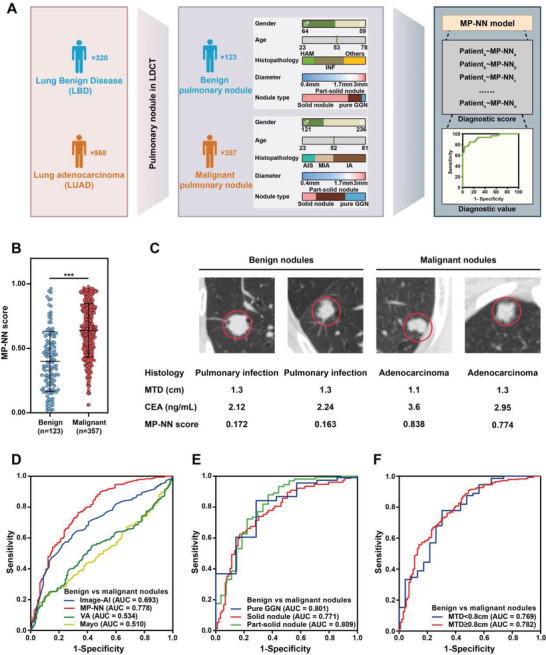
Performance of MP‐NN in pulmonary nodule classification. A) Schematic depicting the approach for evaluation of the performance of MP‐NN in pulmonary nodule classification. B) MP‐NN score levels in patients with benign and malignant nodules. *p*‐values were calculated using a Wilcoxon test. Error bars indicate interquartile. C) Representative data of patients with pulmonary nodules. Left, data of two patients with low MP‐NN scores who were diagnosed with pulmonary infection (# 1486; # 2255). Right, data from two patients with high MP‐NN scores who were diagnosed with lung adenocarcinoma (LUAD) (# 550; # 515). D) Receiver operating characteristic curve (ROC) of MP‐NN, Image‐AI, Mayo Clinic, and Veterans Affairs model for pulmonary nodule classification in the whole cohort. E) ROC of MP‐NN in different nodule radiological subtypes. F) ROC of MP‐NN in different nodule sizes. *** *p* < 0.001.

MP‐NN also works in the classification of pulmonary nodule. A total of 480 samples with pulmonary nodule on LDCT images were selected from the participants (**Figure** **3**A, the detailed demographic characteristics are listed in Table S3, Supporting Information). The average MP‐NN score of patients with malignant pulmonary nodule was significantly higher than that of patients with benign pulmonary nodule (Figure 3B,C). The MP‐NN achieved an AUC of 0.778 (95%CI 0.729–0.828) with sensitivity of 81.79% a specificity of 56.91% (Figure 3D, **Table** **2**). To verify the superiority of MP‐NN model, we introduced the clinical assessment models and CT image artificial intelligence models for comparison. The performance of MP‐NN is superior to the Mayo Clinic and Veterans Affairs models, which are constructed with radiological characteristics and clinical information (*p* < 0.001, Figure 3D). It is worth noting that the performance of MP‐NN was consistent across all nodule sizes, radiological types, and histology types (Figure 3E,F, Table 2). In the intervening years, medical image approaches combined with deep learning technology became a leading research topic in detecting and distinguishing benign and malignant pulmonary nodules.^[^ ^22^ ^]^ We developed a deep learning‐based Image Artificial Intelligence software (Image‐AI) previously.^[^ ^23^ ^]^ As shown in Figure 3D and **Table** **3**, the obtained AUC of MP‐NN was significantly higher than Image‐AI in the test set (*p* = 0.026) and MP‐NN was highly sensitive, but showed poor specificity, while Image‐AI was highly specific, thus a comprehensive intelligent model integrating MP‐NN and Image‐AI will be significantly valuable.

**Table 2:** MP‐NN performance in different nodule sizes and radiological subtypes

Characteristics	AUC (95% CI)	Sensitivity [%]	Specificity [%]	PPV [%]	NPV [%]	Accuracy [%]
**All nodules**	0.778 (0.729–0.828)	81.79	56.91	84.64	51.85	75.42
**Nodule size**						
Nodules ≥ 0.8 cm	0.782 (0.727–0.836)	82.81	57.00	84.59	53.77	76.10
Nodules < 0.8 cm	0.769 (0.653–0.886)	77.78	56.52	84.85	44.83	72.63
**Nodule radiological type**						
Pure GGN	0.801 (0.636–0.966)	87.72	42.86	96.15	17.65	85.12
Part‐solid nodule	0.809 (0.710–0.908)	77.11	70.37	94.12	33.33	76.17
Solid nodule	0.771 (0.698–0.843)	83.12	53.93	60.95	78.69	67.47
**Histological type**						
AIS	0.788 (0.726–0.851)	84.72	56.91	53.51	86.42	67.18
MIA	0.781 (0.722–0.841)	82.52	56.91	61.59	79.55	68.58
IA	0.773 (0.719–0.827)	80.22	56.91	73.37	66.04	70.82

GGN refers to ground‐glass nodule; AIS refers to adenocarcinoma in situ; MIA refers to minimally invasive adenocarcinoma; IA refers to invasive adenocarcinoma.

**Table 3:** Performance of multi‐modal platforms for benign and malignant pulmonary nodule diagnosis

Model	Threshold	AUC (95% CI)	Sensitivity [%]	Specificity [%]	PPV [%]	NPV [%]	Accuracy [%]	*p*1 value^a)^	*p*2 value^b)^
	**Training set**								
**Met‐NN**	0.521	0.717 (0.655–0.778)	82.52	50.00	82.81	49.49	74.22	0.015	<0.001
**Image‐AI**	0.780	0.686 (0.628–0.745)	55.94	76.53	87.43	37.31	61.20	0.128	<0.001
**MP‐NN**	0.433	0.755 (0.698–0.812)	80.77	53.06	83.39	48.60	73.70	/	<0.001
**MPI‐RF**	0.697	0.897 (0.863–0.930)	83.57	80.61	92.64	62.70	82.81	/	/
	**Test set**								
**Met‐NN**	0.521	0.792 (0.682–0.902)	85.92	64.00	87.14	61.54	80.21	0.011	0.008
**Image‐AI**	0.780	0.719 (0.614–0.825)	52.11	84.00	90.24	38.18	60.42	0.026	<0.001
**MP‐NN**	0.433	0.878 (0.792–0.964)	85.92	72.00	89.71	64.29	82.29	/	0.231
**MPI‐RF**	0.697	0.912 (0.854–0.970)	81.69	92.00	96.67	63.89	84.38	/	/

^a)^ The *p*1 values indicate the statistical significance for the differences of AUC comparing the dual modal model MP‐NN with the single modal model Met‐NN and Image‐AI.

^b)^ The *p*2 values indicate the statistical significance for the differences of AUC comparing the tri modal model MPI‐RF with the single modal model Image‐AI and Met‐NN and the dual modal model MP‐NN.

### 2.5 SMFs Based Tri Modal Model for Pulmonary Nodule Classification

**Figure 4:**
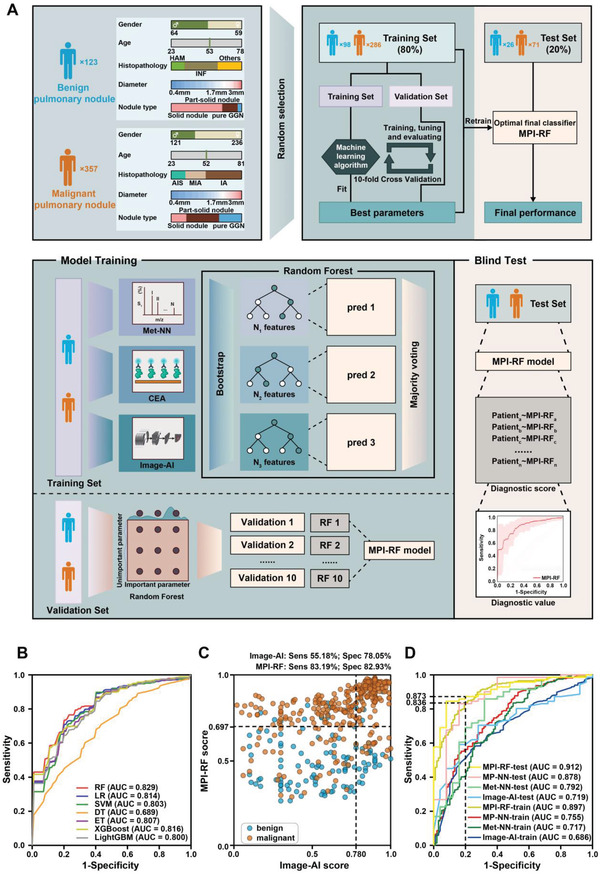
Development and blind test of serum metabolic fingerprints (SMFs) based tri modal pulmonary nodule classification model. A) Schematic overview of the random forest approach used to develop and validate the SMFs based tri modal pulmonary nodule classification model. B) Receiver operating characteristic curve (ROC) of different machine learning algorithms using ten fold cross‐validation in the training set. C) Scatter plot for the graphical comparison of Image‐AI and MPI‐RF in the whole cohort. D) ROC of pulmonary nodule classification models in the training and test set.

To further improve the ability to classify the intermediate pulmonary nodules, we constructed a tri modal model integrating SMFs, tumor marker CEA, and Image‐AI (**Figure** **4**A). To effectively aggregate information from different modalities and select the optimal classifier, we first tested different machine learning models for comparison, including LR, radiological characteristics, SVM, decision trees (DT), extra trees (ET), XGBoost, LightGBM, and RF. The previously 480 samples dataset was randomly split into 8:2 as the training and test set. The RF algorithm was determined to be the best classifier for output fusion based on MP‐NN and Image‐AI on the training set in tenfold cross‐validation (Figure 4B). The best parameters of RF found with grid‐search were adopted to retrain the RF model on the entire training set and generated the final tri modal MPI‐RF model (Figure 4A). As shown in Figure 4C, the integration of Image‐AI with MP‐NN significantly improved the prediction accuracy. MPI‐RF achieved better performance than any single and dual modal models both in the training and the test set (Figure 4D, Table 3). More importantly, at higher specificity (80%), MPI‐RF attained over 80% sensitivity, which is notably better than that of Image‐AI alone (Figure 4D).

Studies have revealed the different proteogenomic landscapes between smokers and non‐smokers.^[^ ^24^ ^]^ We compared smoking status and nodule size to the MPI‐RF score assigned to each sample to assess the effects of clinical risk factors such as smoking and nodule size. The two factors did not appear to correlate with the MPI‐RF score, nodules of all sizes and smoking status were spread across the model score spectrum (Figure S9, Supporting Information). To quantify this observation, the Chi‐square Hosmer and Lemeshow test and Pearson correlation were calculated (Table S4, Supporting Information), and the results indicated that the MPI‐RF was independent of the two currently used risk factors for malignancy (*p* > 0.05). Thus, MPI‐RF model may provide incremental clinical information for pulmonary nodule classification.

## 3 Discussion

LUAD survival is largely dependent on stage at diagnosis. The identification of novel non‐invasive biomarkers is critical to improve the diagnosis of early‐stage LUAD, and is especially important for the accurate classification of pulmonary nodule. A novel lung cancer biomarker will be clinically valuable if it satisfies the unsatisfied clinical need or brings strengths over standard practice (e.g., higher accuracy, more straightforward to use, higher analysis speed, lower costs). Currently, clinically proven lung cancer biomarkers such as CEA are used for monitoring cancer development rather than early diagnosis because of insufficient sensitivity.

Progress in high‐throughput technologies and artificial intelligence approaches has contributed to the discovery of biomarkers for cancer diagnosis and prognosis. Liquid biopsies, such as tumor‐derived autoantibodies,^[^ ^25^ ^]^ circulating tumor DNA methylation panels,^[^ ^26^ ^]^ and protein biomarker panels,^[^ ^27^ ^]^ have been considered as an easier, safer, and less invasive method for cancer diagnosis. However, few approaches have reached extensive clinical use mainly because of methodological limitations. Recently, computer‐aided screening systems have been studied to detect pulmonary nodules and classify malignant and benign ones.^[^ ^5^, ^28^ ^]^ Despite the improved prediction accuracy, several limitations exist, including the issue of overfitting and the lack of clinical/biological information. A single modal marker is unlikely to have sufficient performance because of the heterogeneity and complexity of cancer.^[^ ^29^ ^]^ Hence, identifying new biomarker panels for monitoring cancer presence and progression using advanced omics tools is critical. In this study, we constructed and validated integrative models for lung cancer early detection and pulmonary classification by multi‐modal fusion of radiomics and other omics.

Metabolomics is considered a hallmark of cancer, promising for biomarker development.^[^ ^30^ ^]^ Metabolites are exported from cells to blood and transported out of the body by urine or feces. Thus, metabolites could serve as the efficient and non‐invasive biomarkers which accurately reflect tumor cells' metabolic activity. Current application of metabolites in diagnosis is limited by several critical difficulties including low bio‐molecule abundance and high sample complexity.^[^ ^31^ ^]^ Accordingly, metabolic analysis relies heavily on pretreatment methods, such as chemo‐selective extraction or liquid chromatography, which are time‐intensive and labor‐consuming, making it unpractical for large‐scale use. LDI MS has afforded significant advantages with its rapid and direct analysis of analytes (≈seconds), simple sample preprocessing/preparation for large‐scale use, high sensitivity, and low costs for practical applications.^[^ ^32^ ^]^ Here, we established a rapid NPLDI MS approach to analyze SMFs. This method is distinct from traditional MS approaches that require huge volumes of serum (∼mL) to enhance metabolite abundance and lengthy pretreatment procedures (∼hours) to address sample complexity.

In order to identify accurate and systematic models for LUAD early detection, we further expanded our analysis of SMFs and integrated SMFs with clinical accessible data (e.g., traditional tumor markers or CT features) to generate multi‐modal platforms. We described the development and validation of the dual modal model (MP‐NN) integrating SMFs with tumor marker CEA (Figure 2A), and demonstrated that MP‐NN had better performance than single modal Met‐NN and CEA in screening of early LUAD patients from controls (Figure 2B–H). Of note, the MP‐NN scores correlate well with tumor stage (Figure 2I,J), and may have utility in further study of the treatment monitoring and early detection of cancer relapse. Additionally, our results suggested that the MP‐NN model may be a promising diagnostic adjunct to the current paradigm for detecting indeterminate lung nodules. It achieved an AUC of 0.778 (0.729–0.828, 95% CI), with sensitivity of 81.79% and specificity of 56.91%, which outperformed current clinical prediction models (Figure 3D, Table 2). Recently, a blood‐based methylation model PulmoSeek was demonstrated as a potential solution for lung nodule diagnosis, however, it takes at least 4 days from sample to report, with at least $500 cost (including extraction of cell‐free DNA, conversion of bisulfite, construction of library, sequencing, and bioinformatics analysis) in a clinical laboratory.^[^ ^26b^ ^]^ In contrast, our approach achieved appealing analytical performance (∼seconds) and superior diagnostic performance (AUC of ∼0.8 for pulmonary nodule classification and of ∼0.95 for early LUAD detection), enabling low‐cost and large‐scale rollout for use in clinics. The above results showed that the MP‐NN model provided a new strategy, independent of nodule characters, for accurate classification of pulmonary nodule.

Image features are mostly recommended by guidelines for pulmonary nodule classification, but are limited partly by variability in image interpretation among radiologists and lack of bio‐omics information. The multi‐modal fusion of radiomics and biomics holds great potential to address this issue. Researchers have previously discussed the feasibility of incorporating blood test with routine image scan to improve early cancer screening/diagnosis performance and pulmonary nodule classification.^[^ ^18^, ^23^ ^]^ We constructed a tri modal model integrated SMFs, tumor marker CEA, and Image‐AI via RF classifier (MPI‐RF), which is more robust and accurate for nodule risk stratification than dual modal MP‐NN and single modal Image‐AI (Figure 4). RF is an ensemble tree‐based algorithm involving multiple decision trees which are combined to yield a single prediction that is collective and consensus of multiple trees. By integrating large numbers of decision trees, we can obtain results with dramatic improvements in prediction accuracy. The MPI‐RF model yields an AUC of 0.912 with significantly improved detection sensitivity at higher specificity range than any individual method. This result implicated that radiomics and biomics biomarkers may offer complimentary information and that both domains of knowledge should be utilized for a more comprehensive evaluation of pulmonary nodule classification.

The advantages of our work include the development of the rapid and low‐cost NPLDI MS platform for sensitive and selective collection of SMFs, and the construction of SMFs based single, dual, and tri modal platforms for early LUAD diagnosis and accurate pulmonary nodule classification. This study has several limitations. First, both training and test studies were conducted using retrospective samples, a prospective study is required to further validate the performance of the models. Second, the present study mainly focused on the development and validation of the diagnostic models. The potential pathways and mechanisms need further investigation. Finally, improving lung cancer outcome is the clinic's most important task. We are performing the follow‐up to collect patients' prognostic information, and the outcome of prognostics will be reported in the future.

## 4 Conclusion

Overall, we have developed and validated integrative SMFs based multi‐modal platforms for LUAD early detection and pulmonary nodule classification. The strategy was demonstrated to be practically feasible based on NPLDI MS platform and high‐dimensional data processing methods, and clinically useful with the potential to aid the existing diagnostic approaches in lung cancer screening. This work lays the conceptual and practical foundation for clinical application of omics tools and further provides new insights for biomarker study.

## 5 Experimental Section

### Study Design

A total of 2276 participants, including 320 patients with lung benign disease (LBD), 958 patients with LUAD, and 998 healthy controls (HC) undergoing routine health care maintenance were enrolled between November 2016 and May 2018. The 2276 participants were collected from Shanghai Chest Hospital. All serum samples were collected before or at clinical diagnosis. Exclusion criteria included the lack of histopathologic diagnosis, a history of other types of malignant diseases or acute diseases for patients with lung cancer, and other systematic diseases (e.g., systemic lupus erythematosus) for the controls. Written consent was attained from each participant, with approval by the ethics committee of Shanghai Chest Hospital (ethical approval number KS1961) and registered in the Chinese Clinical Trial Registry (ChiCTR2000036938). Lung cancer was diagnosed by histopathology according to the guidelines of the National Comprehensive Cancer Network.^[^ ^33^ ^]^ Lung cancer staging was performed based on the eighth IASLC TNM Staging System.^[^ ^34^ ^]^ Lung benign diseases included pulmonary infection, chronic obstructive pulmonary disease, hamartoma, and others. There was no significant difference (*p* > 0.05) in age and sex with detailed information listed in Table S1, Supporting Information.

A total of 480 samples were selected from the above participants for multi‐modal pulmonary nodule classification platform construction and validation following the criteria: single pulmonary nodule screened by standard or LDCT with size less than 30 mm; and nodule types of solid nodule, part‐solid nodule, and pure ground‐glass nodule (GGN). The detailed information is listed in Table S3, Supporting Information.

### Sample Size

A power analysis was conducted to decide the significant sample size of machine learning for the MP‐NN and MPI‐RF. Assuming an AUC of 0.95 for MP‐NN was required in the training set, results showed that at least 235 participants (including 99 LUAD patients and 136 controls) would achieve a power of 80% to detect that the true AUC was ≥ 0.9 (1 arm binomial test with one sided alpha = 0.05, Figure S10A, Supporting Information) in the test set. Assuming an AUC of 0.9 for MPI‐RF was required in the training set, results showed that at least 98 participants (including 73 patients with malignant nodules and 25 with benign nodules) would achieve a power of 80% to detect that the true AUC was ≥ 0.8 (1 arm binomial test with one sided alpha = 0.05, Figure S10B, Supporting Information) in the test set.

### Preparation of Serum Sample

Whole blood samples were collected between 6:00 and 7:00 AM, with an overnight fast to remove the disturbance of diet.^[^ ^35^ ^]^ To obtain serum, blood samples were drawn by venipuncture to BD Vacutainer tubes and centrifuged at 3500 rpm for 10 min at 4 °C. Serum samples were stored at −80 °C immediately until use.

### Preparation and Characterization of Nanoparticles

Ferric nanoparticles were synthesized through a modified solvo‐thermal method based on the previous reports.^[^ ^11a^ ^]^ 0.60 g of ferric chloride, 0.15 g of trisodium citrate, and 0.96 g of sodium acetate were dissolved in ethylene glycol solution successively and the mixture was sonicated until the solution became homogeneous. The reaction mixture was heated to 200 °C for 10 h in a reactor with a capacity of 50 mL. The product was washed using deionized water and ethanol multiple times until the supernatant was colorless. The final product was then dried at 60 °C for 12 h and stored in a vacuum before use. For characterization of nanoparticles, SEM images were attained with the S‐4800 (Hitachi, Japan) operating at 10 kV. Transmission electron microscopy images were recorded on a JEM‐2100F instrument (JEOL, Japan) under an acceleration voltage of 300 kV. Dynamic light scattering was conducted on a Nano ZS instrument at 25 °C (Malvern, Worcestershire, UK). Optical absorption measurement of the materials were collected on a UV1900 UV–vis spectrometer (Aucybest, China) at room temperature.

### NPLDI MS Analysis

Ferric nanoparticles were disseminated in deionized water at a concentration of 1 mg mL^−1^ before NPLDI MS detection. An equal volume ratio of serum analytes and material suspension (1 µL) was successively dropped onto the target plates and dried for NPLDI MS test. Mass spectra were acquired in the reflection mode on autoflex speed time‐of‐flight mass spectrometry (Bruker, Germany) with the Nd:YAG laser working at a maximum frequency of 2 kHz and 355 nm. Data from each experiment data were accumulated by 2000 laser shots. Mass calibration was performed with standard molecules to ensure the accurate mass measurement and avoid intra‐plate variation. All serum samples were randomly dropped on multiple 384‐well target plates to reduce systematic errors and inter‐plate variance due to uneven distribution of sample types. In addition, each spot was repeated five times to ensure the reproducibility of the mass spectra.

### Spectrum Preprocessing

The preprocessing of raw MS spectra was performed in a step‐wise manner and included spectrum smoothing, spectrum down‐sampling, baseline correction, and peak detection. For spectrum smoothing, 1D Gaussian Filter with sigma of 1 was performed on the raw spectra to remove noise. For spectrum down‐sampling, the binning operation with a window size of 0.05 Da was performed to reduce the complexity from ≈125 000 data points to 9000 data points. For baseline correction, the white top‐hat operation of the morphological transformations was performed to remove the background baselines. For peak detection, local maxima operation was performed to extract the final metabolic features. After the preprocessing procedures, 2316 metabolic features at the mass range of 100–1000 Da were obtained.

### Tumor Marker Analysis

The serum levels of CEA were measured with a commercially available electrochemiluminescent assay on a Roche Cobas e601 analyzer (Roche Diagnostics, Germany). The cutoff value was set according to the manufacturer's recommendations.

### Existing Clinical Models for Pulmonary Nodule Classification

The Mayo Clinic prediction model for malignancy in pulmonary nodule is described by the following equations:^[^ ^36^ ^]^

(1) $$\mathrm{probability}\;\mathrm{of}\;\mathrm{malignancy}=\frac{e^{x}}{\left(1+e^{x}\right)}$$

(2) $$\begin{array}{ccc} x & = & -6.8272+\left(0.0391\times \mathrm{age}\right)+\left(0.7917\times \mathrm{smoking}\right)\\ & & +\left(1.3388\times \mathrm{cancer}\right)+\left(0.1274\times \mathrm{nodule}\;\mathrm{diameter}\right)\\ & & +\left(1.0407\times \mathrm{spiculation}\right)+\left(0.7838\times \mathrm{upper}\;\mathrm{lobe}\right) \end{array}$$

where *e* refers to the base of natural logarithms, age is the patient's age in years, cigarettes = 0 if the patient has never been a smoker (otherwise = 1, including current or former smoker), cancer = 1 if the patient has a history of extrathoracic cancer which was diagnosed over 5 years ago (otherwise = 0), diameter is the diameter of the pulmonary nodule in millimeters, spiculation = l if pulmonary nodule's edge has spicules (otherwise = 0), and upper = l if the pulmonary nodule is located in an upper lobe (otherwise = 0).

The Veterans Affairs model for malignancy in pulmonary nodule works as the following mathematical equations:^[^ ^5d^ ^]^

(3) $$\mathrm{probability}\;\mathrm{of}\;\mathrm{malignancy}=\frac{e^{x}}{\left(1+e^{x}\right)}$$

(4) $$\begin{array}{ccc} x & = & -8.404+\left(2.061\times \mathrm{smoke}\right)+\left(0.779\times \mathrm{age}10\right)\\ & & +\left(0.112\times \mathrm{diameter}\right)-\left(0.567\times \mathrm{years}\;\mathrm{quit}10\right) \end{array}$$

where *e* refers to the base of natural logarithms, smoke = 0 if the patient has never been a smoker (otherwise 1, including current or former smoker), age 10 refers to the patient's age in years divided by 10, diameter refers to the diameter of the pulmonary nodule in millimeters, and years quit10 is the number of years calculated from the year of quitting smoking divided by 10.

### LDCT Image Artificial Intelligence

CT scans were attained with a row scanner of 128‐detector (produced in Brilliance, Philips, Cleveland, OH, USA) by the helical technique at the end of inspiration throughout one breath hold. Routine CT's scanning parameters were as follows: pitch, 1.0; matrix, 1024 × 1024; FOV, 300 mm; 120 kVp and 200 mA. Deep convolutional neural network model based artificial intelligence software (Image‐AI) for lung nodule detection and classification was developed by Sanmed Biotech and clinical validated previously.^[^ ^23^ ^]^ A 3D‐Unet was used for the nodule detection and segmentation, while downstream tasks including the nodule type classification and cancer risk score prediction network were realized with 3D Resnet. A total of 480 cases of raw chest CT data were transferred to the workstation, and the software system automatically performed lung nodule identification and labeling in batch.

### Diagnostic Model Development

Three models were constructed for LUAD diagnosis and pulmonary nodule classification, including single modal (SMFs), dual modal (SMFs + CEA), and tri modal (SMFs + CEA + CT imaging) based platforms. A neural network was used for the construction of the single modal model based on METabolic fingerprints (Met‐NN) and the dual modal model combined metabolic fingerprints with protein tumor marker CEA (MP‐NN). The single and dual modal classifier consisted of a metabolite‐scoring block and a metabolite‐protein fusion block. The metabolite‐scoring block was designed for calculating the predictive score of metabolic features and consisted of a total of six feature extraction blocks and one fully connected layer for the diagnostic score calculation. Every feature extraction block contained one fully connected layer with 1024 hidden units followed by Dropout operation and LeakyReLU activation. Skip connection based on residual mechanism was introduced to convey information from the input to the output for every feature extraction block. The metabolite‐protein fusion block took the output of the metabolite‐scoring block and the protein tumor marker as the input and then calculated the final probability with one fully connected layer. The Adam optimizer was used with an initial learning rate of 0.0001, *β*_1_ of 0.9 and *β*_2_ of 0.999. Binary cross entropy was applied as the loss function for the guiding of gradient descent. The training process was carried out on a Nvidia GeForce GTX 2070 GPU (Nvidia Corporation, CA, USA) for 1000 epochs. Both Met‐NN and MP‐NN generated a cancer diagnostic score and represented the predictive probability, ranging from 0 to 1. For the Met‐NN and MP‐NN model, the optimal cutoff that maximized the Youden index in the training cohort was applied to estimate the diagnostic specificity, sensitivity, positive predictive value, and negative predictive value. RF was used for the construction of the tri modal (SMFs + CEA + CT imaging) classifier based on the results of MP‐NN and Image‐AI (MPI‐RF). The previous 480 samples dataset was randomly split into 8:2 as the training and test set. On the training set, the RF classifier was trained using ten fold cross‐validation across a grid of values for min_samples_leaf (the minimum number of samples required for a leaf note), max_depth (the maximum depth for one decision tree), and estimators (the number of decision trees in the forest). In each of the ten folds, the RF classifier trained multiple decision trees parallelly with bootstrapping strategy as an ensemble method using 9 of the folds. Every tree was trained with different subsets of samples and features. As a result, each decision tree in the RF was unique. At last, the best parameters found with grid‐search were adopted to retrain the RF model on the entire training set and generate the final tri modal MPI‐RF model. This final MPI‐RF model exhibited good generalization by aggregating the decisions of individual trees. In addition, the resulting MPI‐RF model was blind tested on the remaining test set and compared with MP‐NN and Image‐AI. For the MPI‐RF model, the optimal cutoff that maximized the Youden index in the training cohort was applied to estimate the diagnostic specificity, sensitivity, positive predictive value, and negative predictive value.

### Statistical Analysis

Pre‐processing of mass spectra, machine learning of metabolic fingerprints, and deep learning of metabolic fingerprints and protein tumor marker were performed by home‐build codes with Python (version 3.7), Pytorch (version 1.6.0), Scipy (version 1.5.2), and Sklearn (version 1.0). Statistical analyses (including bio‐analytical results and materials characterizations) were performed using the SPSS software (version 20.0) to calculate the significance (*p*‐value), including Wilcoxon test, Chi‐square Hosmer and Lemeshow test, and Pearson correlation. Binary logistic was carried out by SPSS software (version 20.0). The DeLong test was used to evaluate the significance for the difference of AUCs based on the MedCalc software (version 19.0.4). The PASS software (version 21.0.3) was used to calculate the appropriate sample size. The scatter plots, heat maps, and ROC curves were generated with GraphPad Prism 8.0 (version 8.0) and R (version 3.6.3) package “ROCR” (version 1.0‐11) and then embellished with Adobe Illustrator (version 23.0).

## Conflict of Interest

The authors have filed patents for both the technology and the use of the technology to analyze biosamples.

## Author Contributions

L.W., Me.Z., X.P., and Mi.Z. contributed equally to this work. K.Q. and J.L. planned this work and designed the overall approach with L.W., Me.Z., X.P., and Mi.Z. L.W., X.P., Mi.Z., L.H., and X.H. carried out experiments and wrote the manuscript. Me.Z. performed the preprocessing of mass spectra, deep learning, and machine learning of the metabolic and proteomic data. Mi.Z., X.W., L.Q., Q.G., W.X., W.Q., M.L., and H.S. helped with sample collection. T.X. and Xia.Y. contributed to the CT analysis. Y.K., Xin.Y., and X.L. contributed to the machine learning analysis of medical images. All authors discussed the results and reviewed the manuscript.

## Supporting information

Supporting Information

## Data Availability

The data that support the findings of this study are available from the corresponding author upon reasonable request.
