# Supplementary material for: Integrative Serum Metabolic Fingerprints Based Multi‐Modal Platforms for Lung Adenocarcinoma Early Detection and Pulmonary Nodule Classification
Source: Adv Sci (Weinh). 2022 Oct 18;9(34):2203786. doi: 10.1002/advs.202203786 (PMC9731719; doi:10.1002/advs.202203786)
Supplement: Supplementary file 1 — Supporting Information [file ADVS-9-2203786-s001.pdf]

ADVANCED SCIENCE
Open Access

Supporting Information

for *Adv. Sci.*, DOI 10.1002/advs.202203786

Integrative Serum Metabolic Fingerprints Based Multi-Modal Platforms for Lung Adenocarcinoma Early Detection and Pulmonary Nodule Classification

Lin Wang, Mengji Zhang, Xufeng Pan, Mingna Zhao, Lin Huang, Xiaomeng Hu, Xueqing Wang, Lihua Qiao, Qiaomei Guo, Wanxing Xu, Wenli Qian, Tingjia Xue, Xiaodan Ye, Ming Li, Haixiang Su, Yinglan Kuang, Xing Lu, Xin Ye, Kun Qian and Jiatao Lou**

Supporting Information

Integrative Serum Metabolic Fingerprints Based Multi-modal Platforms for Lung Adenocarcinoma Early Detection and Pulmonary Nodule Classification

Lin Wang, Mengji Zhang, Xufeng Pan, Mingna Zhao, Lin Huang, Xiaomeng Hu, Xueqing Wang, Lihua Qiao, Qiaomei Guo, Wanxing Xu, Wenli Qian, Tingjia Xue, Xiaodan Ye, Ming Li, Haixiang Su, Yinglan Kuang, Xing Lu, Xin Ye, Kun Qian, Jiatao Lou**

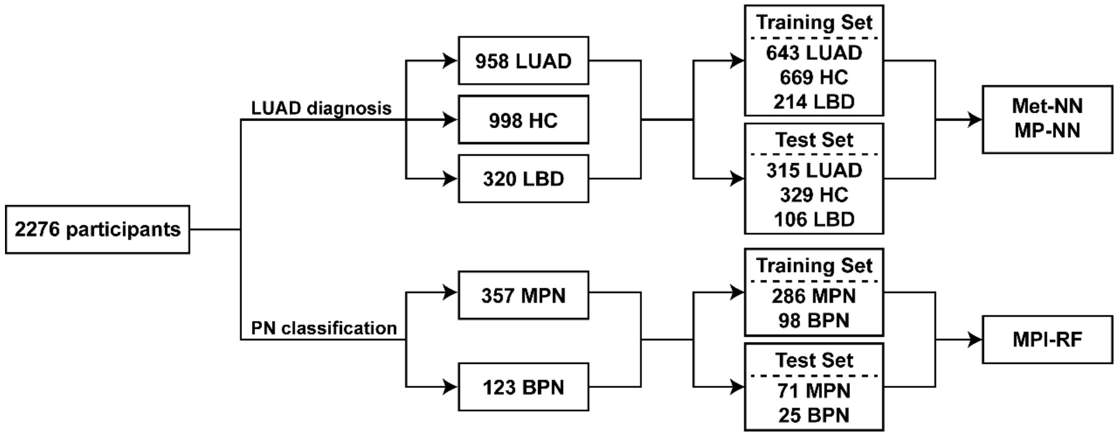

Figure S1. Schematic flow of participants in the study.

LUAD = Lung Adenocarcinoma. HC = Healthy Control. LBD = Lung Benign Disease. BPN = Benign Pulmonary Nodule. MPN = Malignant Pulmonary Nodule. Met-NN = Single modal model based on serum metabolic fingerprints (SMFs) by neural network. MP-NN = Dual modal model combining SMFs with protein tumor marker CEA by neural network. MPI-RF = Tri modal model integrating SMFs, protein tumor marker CEA and image features by random forest.

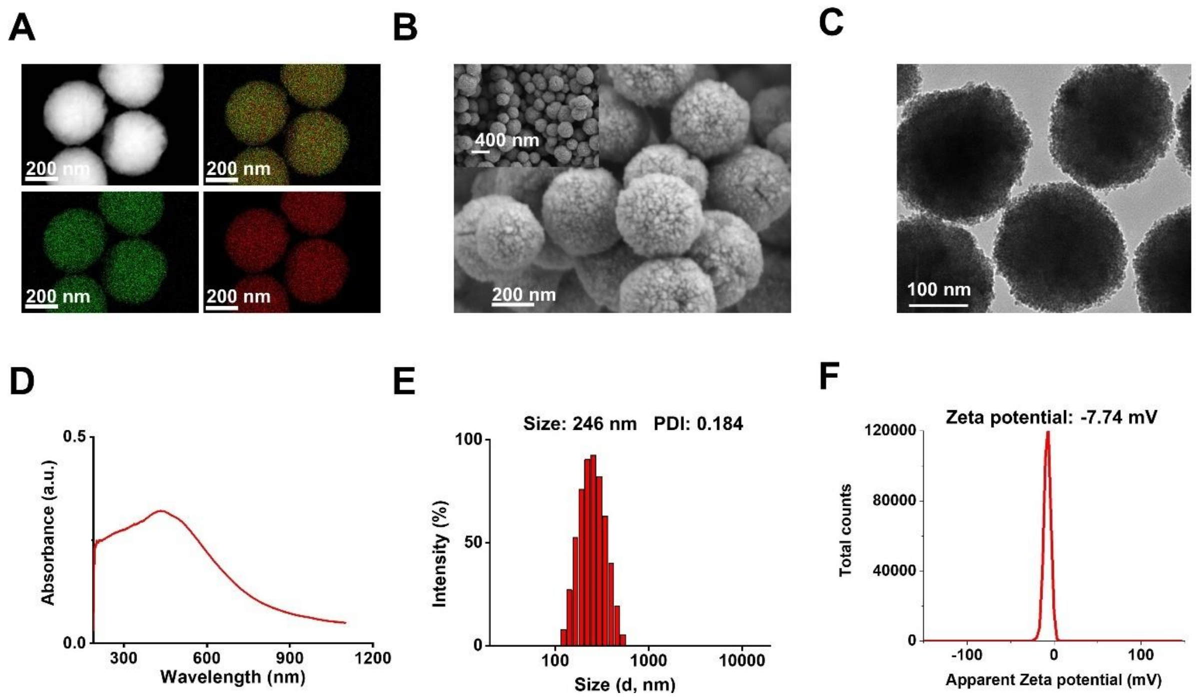

Figure S2. Characterization of ferric nanoparticles.

(A) Elemental mapping, (B) scanning electron microscopy (SEM) images, and (C) transmission electron microscopy (TEM) image of ferric nanoparticles. (D) Ultraviolet-visible (UV-vis) analysis of ferric nanoparticles. (E) Size distribution of ferric nanoparticles at 25°C in water by dynamic light scattering (DLS). (F) Zeta potential distribution of the ferric nanoparticles.

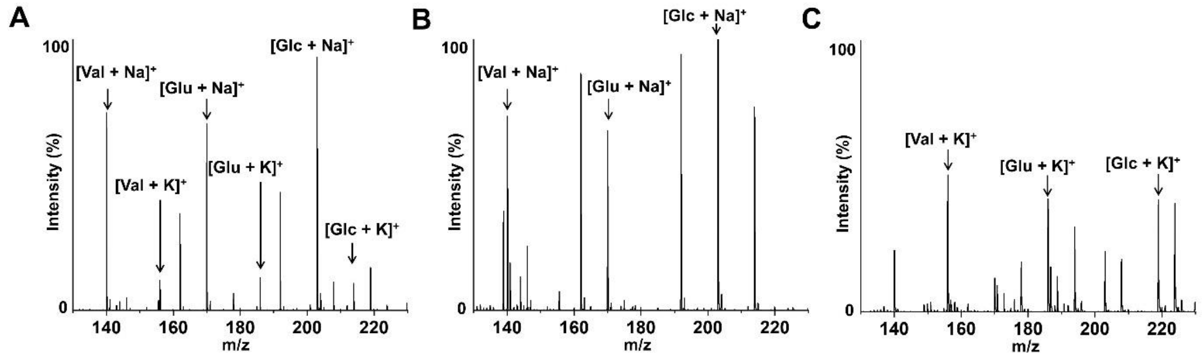

Figure S3. Protein/salt tolerance of NPLDI MS.

Mass spectra from NPLDI MS using samples of (A) 2 nmol valine (Val), glutamic acid (Glu), and glucose (Glc) in 5 mg/mL bovine serum albumin solution, (B) 2 nmol valine, glutamic acid, and glucose in 0.5 M NaCl solution, and (C) 2 nmol valine, glutamic acid, and glucose in 0.5 M KCl solution. All the results were recorded in positive ion mode, and Na+ /K+ adducts were observed.

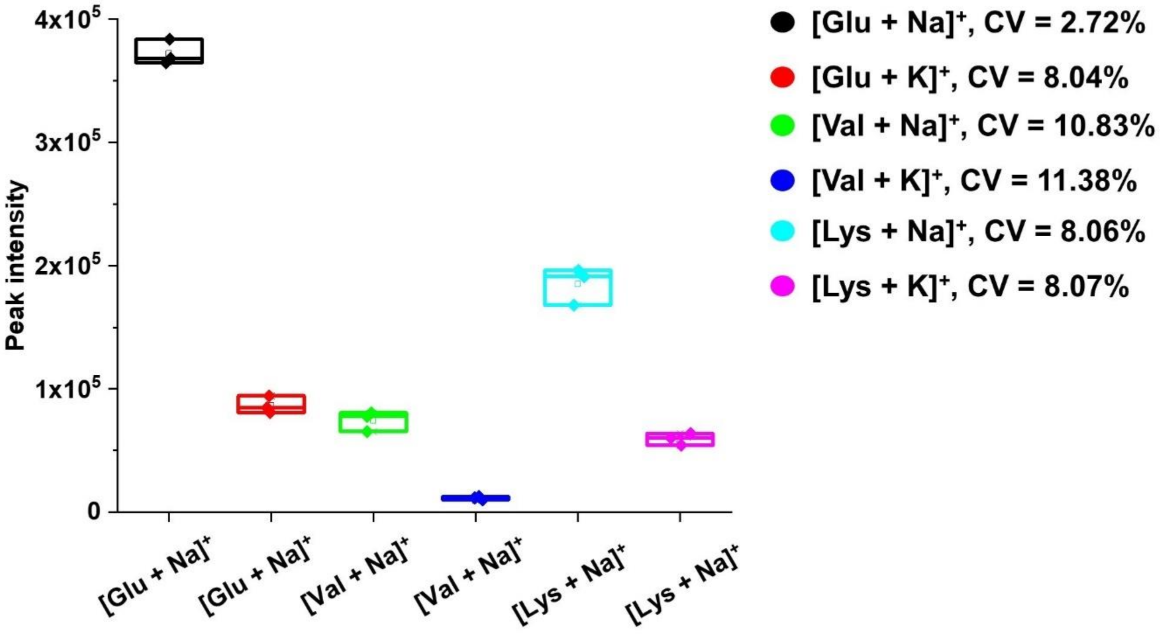

Figure S4. Results of NPLDI MS detection in three technical replicates.

Intensities of glucose (Glu, 1 ng/nL) of 1 μL, valine (Val, 1 ng/nL) of 1 μL, and lysine (Lys, 1 ng/nL) of 1 μL using ferric nanoparticles in three technical replicates.

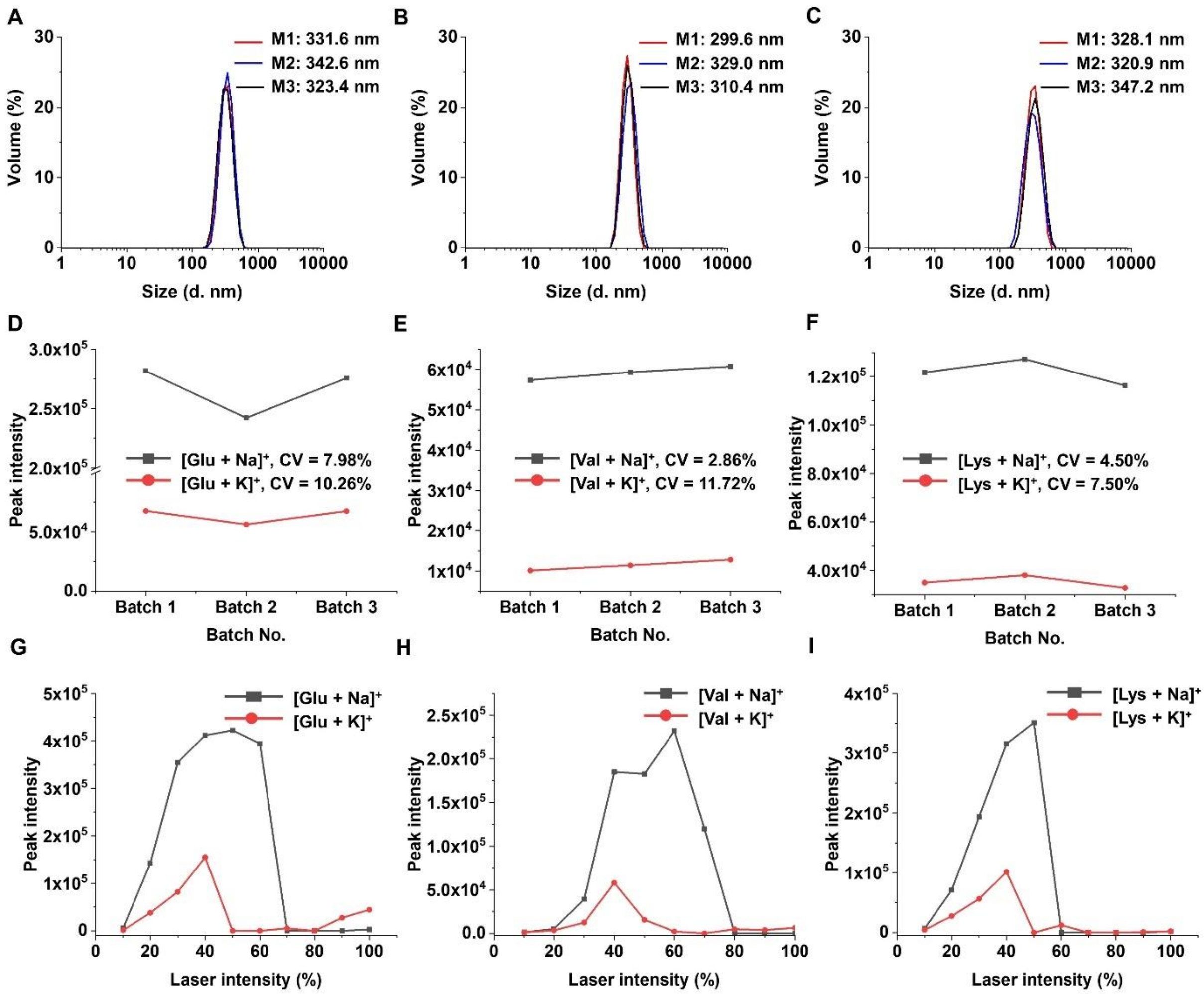

Figure S5. NPLDI MS detection using nanoparticles from three batches and using different laser power.

Size distribution of ferric nanoparticles from (A) batch 1, (B) batch 2, and (C) batch 3 by dynamic light scattering. Detection results of (D) glucose (Glu, 1 ng/nL) of 1 μL, (E) valine (Val, 1 ng/nL) of 1 μL, and (F) lysine (Lys, 1 ng/nL) of 1 μL using ferric nanoparticles from different batches. LDI MS of glucose, valine, and lysine using different laser intensity. Detection results of G) glucose (Glu, 1 ng/nL) of 1 μL, (H) valine (Val, 1 ng/nL) of 1 μL, and (I) lysine (Lys, 1 ng/nL) of 1 μL using ferric nanoparticles in positive mode.

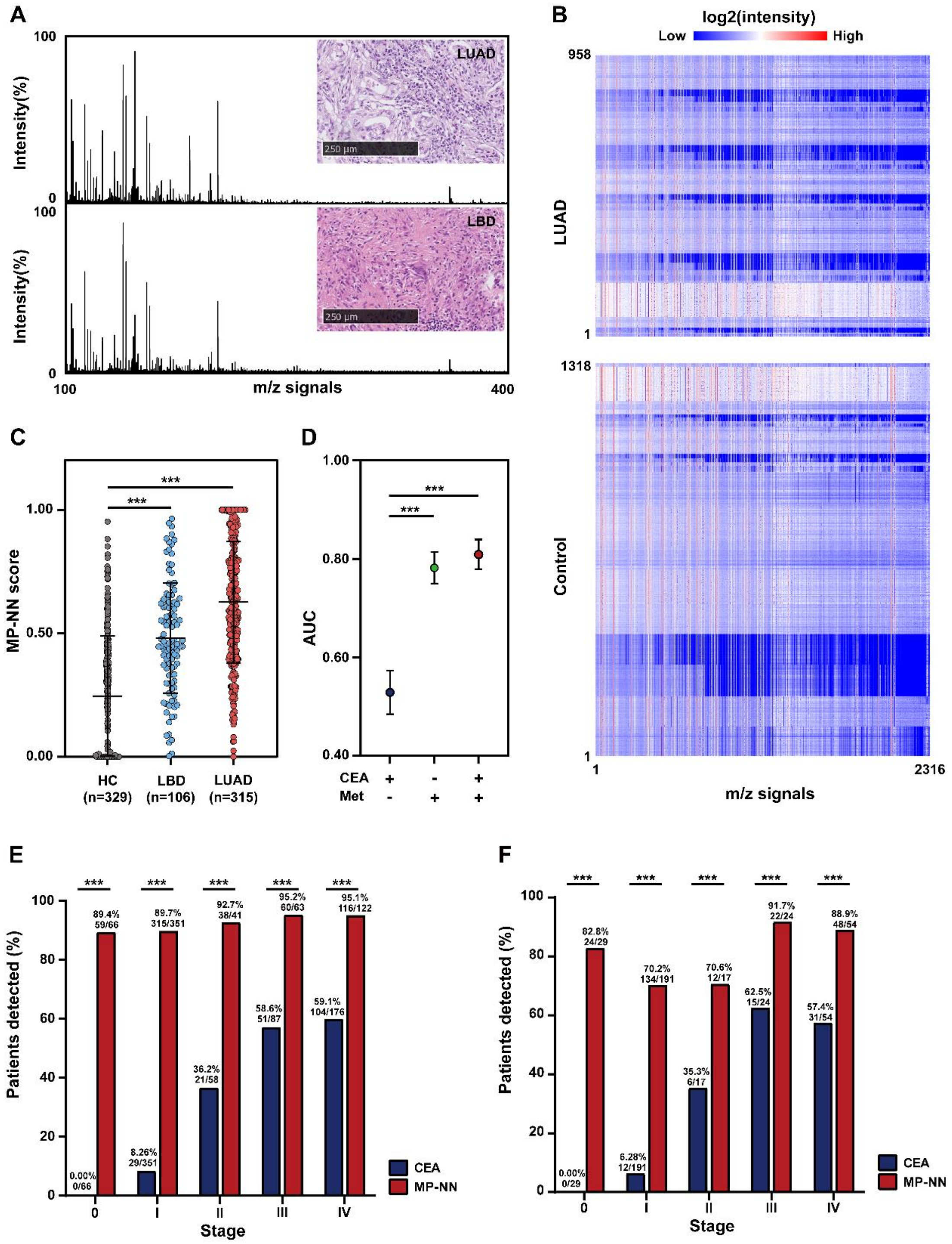

Figure S6. Serum metabolic database construction and performance of MP-NN.

(A) Typical metabolic mass spectrum of LUAD and LBD (granuloma). Insets show the optical images of the H&E-stained tissue sections evaluated by histopathology. (B) Extracted serum metabolic fingerprints. (C) Score of the metabolic signature identified in the test cohort. *p* values were calculated using a Wilcoxon test. Error bars indicate interquartile. (D) AUC for individual parameters in the test cohort. *p* values were calculated using a DeLong test. Error bars indicate 95% confidence intervals (CIs). (E) MP-NN detection rates summarized by

stage in the training cohort. *p* values were calculated using a Chi-square test. (F) MP-NN detection rates summarized by stage in the test cohort. *p* values were calculated using a Chi-square test. ***$p < 0.001$.

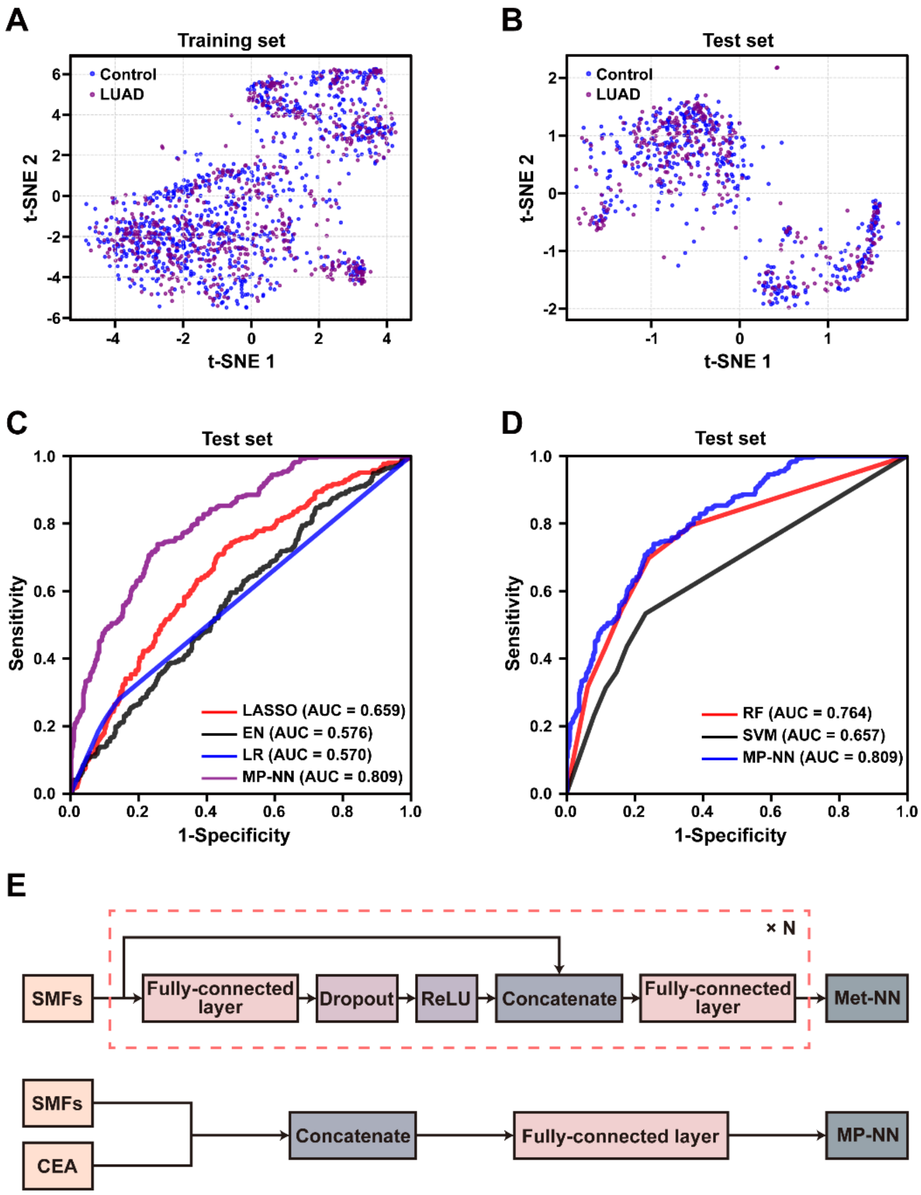

Figure S7. Diagnostic performance of MP-NN.

(A and B), t-SNE analysis of SMFs. Purple for the LUAD patients and blue for the controls (HC and LBD) in (A) training set and (B) test set. (C) Diagnostic performance by non-linear model (MP-NN) and linear models (elastic net (EN), least absolute shrinkage and selection operator (LASSO), and logistic regression (LR)) in blind test set. (D) Diagnostic performance by MP-NN (handling of unstructured data) and support vector machine (SVM) and random forest (handling of structured data). (E) The network architecture for construction of Met-NN and MP-NN via artificial neural network.

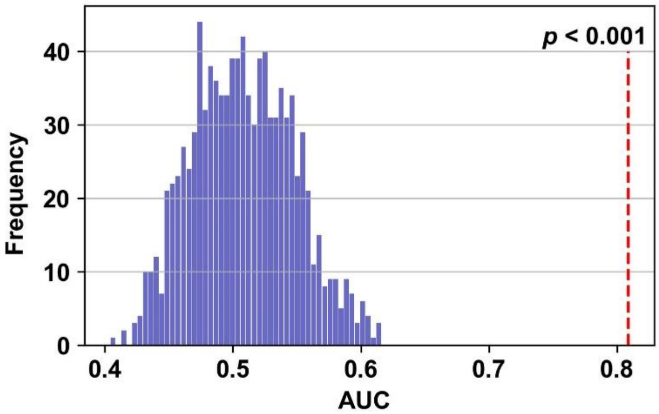

Figure S8. **Permutation test of the deep learning model MP-NN.**

Distribution of test AUC computed by the uninformative metabolic features obtained by random permutation (1,000 permutations).

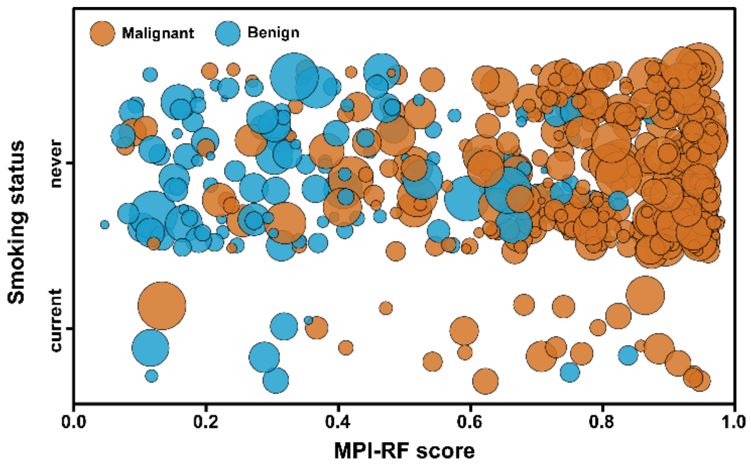

Figure S9. Correlation analysis of clinical factors (smoking status and nodule size) with MPI-RF score.

Multivariate analysis of clinical factors and MPI-RF scores as they relate to malignant and benign samples in the training and test set. Nodule size is represented by circle diameter.

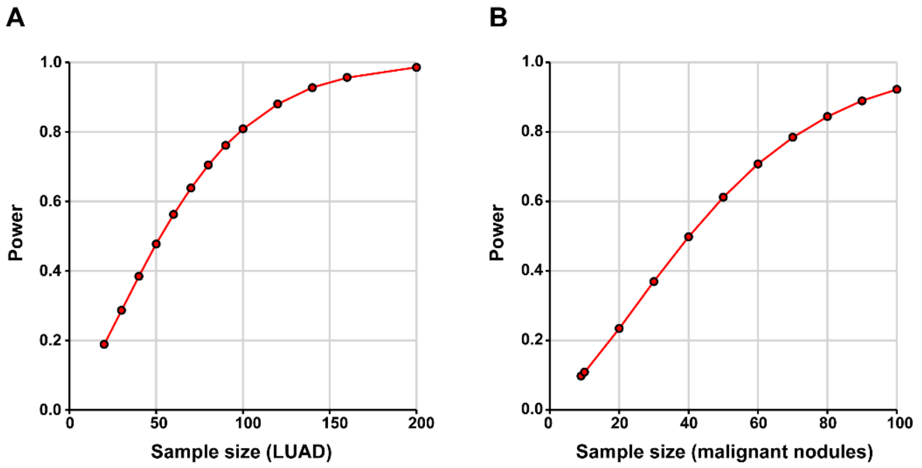

Figure S10. Power analysis for (A) the detection of LUAD and (B) the classification of pulmonary nodule.

Table S1. Clinical characteristics of the enrolled participants.

Characteristics	Training Set			Test Set		
	LUAD (n = 643)	Controls (n = 883)	*p* value [a)]	LUAD (n = 315)	Controls (n = 435)	*p* value [a)]
Age, years	56 (48.5-64)	58 (42-67)	0.878	55 (48-64)	60 (46-66)	0.105
Gender, n						
Male	265	368	0.856	105	168	0.137
Female	378	515		210	267	
Histology						
LUAD	643	—		315	—	
HC	—	669		—	329	
LBD						
INF	—	119		—	61	
COPD	—	28		—	8	
HAM	—	17		—	8	
Others	—	50		—	29	
Stage						
0	66	—		29		
I	351	—		191	—	
II	41	—		17	—	
III	63	—		24	—	
IV	122	—		54	—	

Data are n, or median (range), unless otherwise specified. LUAD = lung adenocarcinoma. HC = healthy control. LBD = lung benign disease. INF = pulmonary infection. COPD = chronic obstructive pulmonary disease. HAM = hamartoma. [a)] The *p* values indicate the statistical significance among patients and controls.

Table S2. Detection limit of standard metabolites by NPLDI MS.

Analytes	Detection-of-limit (pmol)
Glucose	28
Sucrose	1.5
Cellobiose	1.5
Mannitol	2.7
Leucine	38
Lysine	34
Methionine	34
Glutamic acid	34
Arginine	29

Table S3. Clinical characteristics of the enrolled participants with pulmonary nodule in CT.

Characteristics	Training Set			Test Set		
	Patients with malignant nodules (n = 286)	Patients with benign nodules (n = 98)	*p* value [a)]	Patients with malignant nodules (n = 71)	Patients with benign nodules (n = 25)	*p* value [a)]
Age, years	52.5 (47-60.8)	52 (45-60)	0.331	54 (51-60)	51 (43-58.5)	0.095
Gender, n						
Male	100	53	0.001	21	11	0.188
Female	186	45		50	14	
Smoking status						
Never	267	92	0.857	68	24	0.961
Current	19	6		3	1	
Nodule size						
< 0.8 cm	58	17	0.527	14	6	0.650
≥0.8 cm	228	81		57	19	
Nodule location						
LUL	73	22	0.657	24	4	0.003
LLL	47	16		6	8	
RUL	89	27		23	5	
RML	20	11		3	5	
RLL	57	22		15	3	
Nodule type						
Pure GGN	88	5	< 0.001	26	2	< 0.001
Part-solid	134	20		32	7	

Solid	64	73		13	16	
Spiculation						
Yes	50	11	0.144	12	3	0.562
No	236	87		59	22	
Histopathology						
AIS	53	—		19	—	
MIA	86	—		17	—	
IA	147	—		35	—	
INF	—	44		—	14	
HAM	—	20		—	3	
Others	—	34		—	8	

Data are n, or median (range), unless otherwise specified. LUL = left upper lobe. LLL = left lower lobe. RUL = right upper lobe. RML = right middle lobe. RLL = right lower lobe. GGN = ground-glass nodule. AIS=adenocarcinoma in situ. MIA = minimally invasive adenocarcinoma. IA = invasive adenocarcinoma. INF = pulmonary infection. HAM = hamartoma. [a)] The *p* values indicate the statistical significance among patients with benign and malignant nodules.

Table S4. Analysis of Correlation between MPI-RF score and smoking status or nodule size.

Risk factor	All samples	*p* value	Malignant samples	*p* value	Benign samples	*p* value
Smoking status	0.002	0.759 [a)]	0.007	0.703*	<0.001	0.305 [a)]
Nodule size (mm)	0.004	0.926 [b)]	0.009	0.873**	-0.040	0.658 [b)]

[a)] *p* value of Hosmer and Lemeshow Test for Binary logistic. [b)] *p* value of Pearson correlation
